# Fungal–Metal Interactions: A Review of Toxicity and Homeostasis

**DOI:** 10.3390/jof7030225

**Published:** 2021-03-18

**Authors:** Janelle R. Robinson, Omoanghe S. Isikhuemhen, Felicia N. Anike

**Affiliations:** Department of Natural Resources and Environmental Design, North Carolina Agricultural and Technical State University, 1601 East Market Street, Greensboro, NC 27411, USA; jrrobin3@aggies.ncat.edu (J.R.R.); fnanike@ncat.edu (F.N.A.)

**Keywords:** resistance, homeostasis, toxicity, nanoparticles, fungal–metal interaction

## Abstract

Metal nanoparticles used as antifungals have increased the occurrence of fungal–metal interactions. However, there is a lack of knowledge about how these interactions cause genomic and physiological changes, which can produce fungal superbugs. Despite interest in these interactions, there is limited understanding of resistance mechanisms in most fungi studied until now. We highlight the current knowledge of fungal homeostasis of zinc, copper, iron, manganese, and silver to comprehensively examine associated mechanisms of resistance. Such mechanisms have been widely studied in *Saccharomyces cerevisiae*, but limited reports exist in filamentous fungi, though they are frequently the subject of nanoparticle biosynthesis and targets of antifungal metals. In most cases, microarray analyses uncovered resistance mechanisms as a response to metal exposure. In yeast, metal resistance is mainly due to the down-regulation of metal ion importers, utilization of metallothionein and metallothionein-like structures, and ion sequestration to the vacuole. In contrast, metal resistance in filamentous fungi heavily relies upon cellular ion export. However, there are instances of resistance that utilized vacuole sequestration, ion metallothionein, and chelator binding, deleting a metal ion importer, and ion storage in hyphal cell walls. In general, resistance to zinc, copper, iron, and manganese is extensively reported in yeast and partially known in filamentous fungi; and silver resistance lacks comprehensive understanding in both.

## 1. Introduction

The increasing applications of fungal–metal interactions have led to the need for research on their contributions to fungal resistance [1,2]. In nature, metals serve as micronutrients required for fungal growth, however, in excess they can influence homeostatic systems. In agricultural and human medicine, there is an increasing occurrence of pathogen resistance to traditional antifungal agents which has expanded the incidence of fungal superbugs; this has led to increased research on metals as alternative fungistatic and fungicidal agents [3,4]. Fungi are also being employed in the green biosynthesis of nanoparticles due to their economic viability, high levels of natural metal resistance, and ease of mass production as antimicrobial agents [5,6,7]. Both instances highlight contributions to increased incidence of fungal-metal interactions, demonstrating the importance of further divulging the intricacies of their relationship.

### 1.1. Fungal–Metal Interactions

Metals can exist in various forms such as salts, oxides, sulfates, and nanoparticles. Fungi are able to utilize metal ions from these compounds after dissociation, which leaves unbound ions available for uptake and transport. For example, in the presence of water, copper sulfate (CuSO_4_) hydrates to copper (II) sulfate pentahydrate (CuSO_4_ 5H_2_O) and then dissociates into Cu^2+^ + SO_4_^2−^. Upon dissociation, Cu^2+^ can then be reduced by fungal proteins for uptake. More recently, metals in the form of nanoparticles have gained interest for use as antifungals, which has fueled the escalation of nanoparticle production [8,9,10]. Nanoparticles are particles that range from 1 to 100 nm in size and vary in shape, physiochemical, optical, and biological properties [11]. Ions dissociate from nanoparticles at a much lower rate, but are also available to interact with homeostatic systems [12,13].

**Figure 1:**
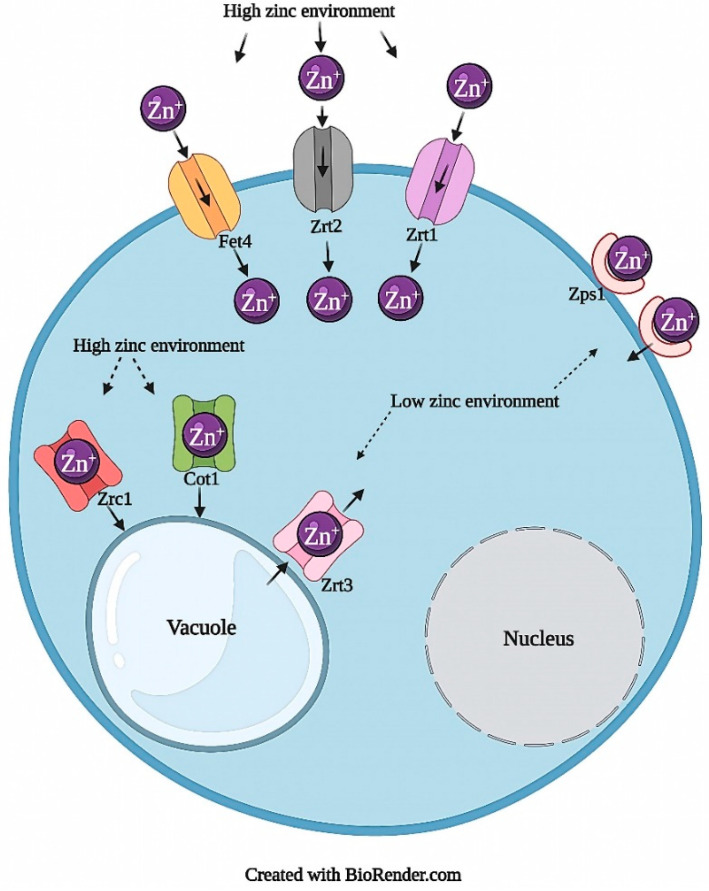
*S. cerevisiae* zinc homeostatic systems.

**Figure 2:**
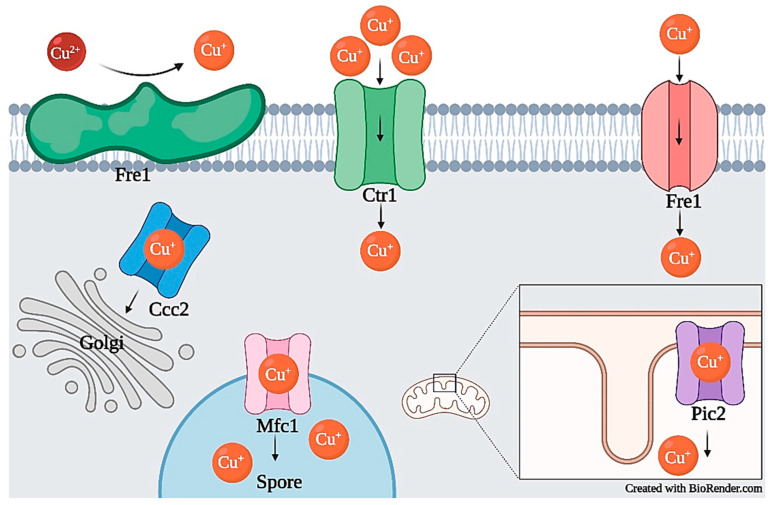
Yeast copper transport systems. In *S. cerevisiae*, cupric reductase, Fre1 reduces extracellular cupric oxide for transport across high and low-affinity copper membrane transports Ctr1 and Fet4. From the cytoplasm, Ccc2 shuttles Cu^+^ to Golgi bodies, and Pic2 shuttles Cu^+^ to the mitochondrial matrix. During meiosis in *S. pombe*, Mfc1 transports Cu^+^ across the forespore membrane.

In general, most ions have dedicated homeostatic systems to control import, export, storage, and transport within the cell (Table 1). Metal ion import and export often occurs through transmembrane channels, which are proteins that span the entirety of the membrane and protrude from both sides (e.g., transmembrane proteins Fet4, Zrt1, and Zrt2 in Figure 1) [14,15]. In some species, chelators, such as siderophores, also play a role in uptake. These organic, low molecular weight compounds have a binding capacity for certain metal ions, such as iron, and are imported into the cell through transmembrane channels [16,17]. As a mechanism of ion storage or detoxification, metallothioneins (MTs), cysteine-rich proteins that use metal ions as cofactors, bind free cytosolic ions which may be released back into the cellular environment in metal deficient conditions [18,19]. For the movement of ions to organelles for storage or as cofactors for protein functioning, intracellular transporters, such as Zrc1 (Figure 1) or Pic2 (Figure 2), are utilized [20,21]. If these systems are interfered with, homeostatic imbalance can cause toxicity.

**Table 1:** Fungal proteins involved in metal transport.

Metal	Transport Type	Yeast Transporters	Reference	Filamentous Fungi Transporters	Reference
Zinc	Import	Zrt1, Zrt2	[15,22]	*zrfA/B/C*, UmZRT1/2, Zip1/2	[23,24,25,26,27]
	Vacuolar	Cot1, Zrc1	[20,28]	-	-
	Vacuole to Cytosol	Zrt3	[29]	-	-
Copper	Import	Ctr1, Ctr3, Fet4, Ctr4, Ctr5, Mfc1	[30,31,32,33,34,35,36]	CtrA2, CtrC, Ctr1, PaCtr2	[37,38,39]
	Cytosol to Golgi	Atx1, Ccc2	[34,40,41,42]	-	-
	Mitochondrial	Pic2, Cox17	[21,43,44]	-	-
	Cytosol to Sod1	Lys7, Pccs	[45,46]	-	-
	Mitochondrial Inner Membrane Space to Cytochrome *c* oxidase	Sco1, Sco2, Cox11	[42,47,48]	-	-
	Export	-	-	CrpA	[49]
Iron	Import	Fet4, Smf1, Fet3/Ftr1, Fip1, Str3, Shu1, Str1, Str2, Str3	[50,51,52,53,54,55,56,57,58,59]	Fer2	[60]
	Within the Nucleus	Npb35, Nar1, Cfd1, Cia1	[61,62]	-	-
	Vacuolar	Pcl1, Ccc1	[63,64]	-	-
Mangan-ese	Import	Smf1, Smf2, Pho85	[52,65,66,67]	PcPho84, PcSmfs	[68]
	Mitochondrial	Mtm1	[69]	PcMtm1	[68]
	Cytosol to Golgi Lumen	Pmr1, Gdt1	[70,71,72]	-	-
	Cytosol to Endoplasmic Reticulum Lumen	Spf1	[73]	-	-
	Vacuolar	Ccc1, Ypk9	[64,74,75,76]	PcCCC1	[68]
	Export	Pmr1, Hip1	[77,78,79]	PcMnt	[68]
Silver	Import	Ctr1	[80,81]	-	-
	Mitochondrial	Pic2	[21]	-	-

### 1.2. Metal Toxicity and Resistance

Metal toxicity occurs via the oligodynamic effect, which was initially described in 1893 in algae *Spirogyra nitida* and *Spirogyra dubia*, as toxicity or death in organisms due to exposure to trace amounts of metals, such as copper, lead, iron, or zinc [82]. In fungi, this exposure can have effects ranging interference in ergosterol biosynthesis to reduced MT activity (Table 2) [83,84].

**Table 2:** Mechanisms of toxicity in yeast and filamentous fungi.

Metal	Mechanism of Toxicity in Yeast	Reference	Mechanism of Toxicity in Filamentous Fungi	Reference
Zinc	Interference of synthesis of iron-sulfur clusters	[85,86]	increased chitin deposition within the cell wall, preventing hyphal extension	[87,88]
	Interference in ergosterol biosynthesis	[83]	increased hyphal branching and apical swelling	[88]
	Cellular leakage, polarization, and increased membrane potential	[83]	interruption of conidia and conidiophore development (interference of reproduction)	[87]
	Reduced cell wall integrity	[83]	-	-
Copper	Reduced ergosterol biosynthesis	[12,89]	Generation of reactive oxygen species	[90]
	Reduced metallothionein activity	[84]	-	-
Iron	Interference of vacuolar transport encoding gene *CCC1*	[91,92]	Inability to acquire iron	[60,93]
Manganese	Down-regulation of *HTB2*, *HTA1*, *HTA1*, *HTBI*, *HHF*	[94,95]	potentially associated to reduced functioning of manganese peroxidase	[96,97,98]
Silver	Interference in ergosterol biosynthesis	[80,99,100]	-	-

In an effort to counter metal toxicity, and toxicity in general, fungi develop methods of resistance which can include the alteration of the target protein to inhibit substrate binding, cellular antimicrobial efflux, antimicrobial inactivation or degradation, restricted uptake to prevent cellular interference, overproduction of targeted proteins to prevent the complete inhibition of biochemicals, and compensation for loss of function directly related to the antimicrobial [101]. Some of these resistance mechanisms are relevant to excessive metal exposure in fungi (Table 3). Presently, research utilizes yeast such as *Saccharomyces cerevisiae* to investigate cellular and molecular impacts of fungal–metal interactions, but thorough knowledge is lacking in filamentous fungi [102,103,104]. Due to the increase in fungal-metal interactions, we should ensure that metal resistance mechanisms in multiple types of fungi are well-understood. In this review, we summarize existing knowledge on fungal metal homeostasis of zinc, copper, iron, manganese, and silver. Conclusions and indications are presented to pave the way for further research.

**Table 3:** Mechanisms of metal resistance in yeast and filamentous fungi.

Metal	Mechanism of Metal Resistance in Yeast	Reference	Mechanism of Metal Resistance in Filamentous Fungi	Reference
Zinc	Up-regulation of *ZRC1* and *COT1*	[83,105,106,107,108]	storage of excess zinc in vacuoles and cell walls of spores and hyphae	[109,110]
	-	-	zinc efflux	[111]
	-	-	zinc metallothioneins	[112]
Copper	Up-regulation of *CUP1* and *CRS5*	[113]	Up-regulation of *crpA*	[81,114,115,116]
	Down-regulation of *FRE1* and *FRE7*, and *CTR1*	[113]	increased production of chelator copper oxalate	[117,118,119]
Iron	Up-regulation of *CCC1*	[64,120]	Unknown, but could associated with reduction of siderophore biosynthesis	[60,121]
	Expression of plant ferritin genes	[122,123,124]	-	-
Manganese	Up-regulation of *MNR1*	[65,67,125,126]	Deletion of *PcPHO84*	[68]
	Down-regulation of *PHO84*, *SMF1*	[67,125,126]	Expression of *PcMNT*	[68]
Silver	Expression of *CUP1-1*, *CUP1-2*	[81,115,116]	Expression of *crpA*	[90]
	Down-regulation of *PHO84*	[116]	-	-

## 2. Fungal–Metal Interactions

Metals play critical roles in fungal homeostasis. They are required for various biochemical processes, usually as enzymatic cofactors. Metals most recognized for their importance in fungi are copper, iron, zinc, and manganese. Pertaining to zinc, approximately 5% of fungal proteomes correlate to zinc-binding proteins, and 8% of yeast genomes correlate to zinc-binding proteins. In the model yeast *S. cerevisiae*, large portions of these zinc-binding proteins are related to critical functions, including DNA binding (31% of zinc-binding proteins), the regulation of transcription (25%), transcription factor activity (19%), and response to chemical stimuli (15%) [105,107,108]. Fungal–copper interactions are necessary for the activation of metalloproteins involved in biochemical processes. This includes the activation of superoxide dismutase, which is responsible for cellular detoxification of reactive oxygen species (ROS), virulence in pathogenic species, and activation of cytochrome *c* oxidate, a catalyst within the electron transport chain [39,48]. Iron is also essential for fungal virulence in pathogenic species, most importantly as an integral component of iron-sulfur clusters which are required for the activation of nuclear proteins involved in DNA repair [61]. Manganese also plays a critical role in fungi, in particular, in filamentous species where it (or copper) is required for the activation of manganese peroxidase. Dependent on nutrient availability, white-rot fungi utilize manganese peroxidase as a secondary metabolite to depolymerize lignin for nutrients; others are manipulated for increased manganese peroxidase production and extraction for use in the degradation of organo-pollutants [96,98].

Very few metals that are not considered essential have also been identified in some fungal–metal interactions; these include magnesium and molybdenum. Magnesium is a well-known micronutrient in other eukaryotic organisms, however, its homeostasis in fungi is undetermined. Only in recent years has magnesium been identified as a requirement for virulence in the agriculturally relevant fungus *Magnaporthe oryzae* [127]. Molybdenum is a metal that is discussed significantly less in eukaryotic homeostasis. It has only been identified as a cofactor for four human proteins, and in fungi it has only been suggested that it plays an unidentified role as a nitrate reductase and a xanthine dehydrogenase [128,129]. Other metals such as silver, gold, lead, nickel, and cadmium have only been implicated in fungal–metal interactions related to toxicity, nanoparticle myco-synthesis, and heavy metal myco-remediation, but information pertaining to homeostasis is limited [103,130,131].

### 2.1. Zinc

Zinc is a transition metal required for fungal survival and is necessary for various functions, including the structuring of nucleic acids, physical growth and, most predominately, protein folding [132,133]. In its role in DNA binding, zinc presents itself in class III zinc finger proteins, also known as zinc cluster proteins (Zn(II)_2_Cys_6_), found only in Ascomycetes (with the singular exception of *Lentinus edodes*) [107,134,135,136]. This protein class binds DNA, which is critical for the transcriptional activation and regulation of gene products [105,134].

In agriculture, fungal infections threaten food security by increasing global crop loss [137,138]. Traditionally, antifungal azoles have been used to combat disease, but with the emergence of azole-resistant pathogens, scientists have begun to develop possible alternatives, such as zinc-containing compounds [138,139]. Reports have demonstrated that zinc oxide nanoparticles (ZnO NPs) can control postharvest mold, plant wilts, and grey mold disease caused by *Aspergillus niger*, *Fusarium oxysporum*, and *Botrytis cinerea*, respectively [7,140,141,142,143]. It has also been demonstrated that ZnO NPs can significantly reduce the production of the mycotoxin fusaric acid from *F. oxysporum* [144]. This is significant because mycotoxins are common secondary metabolites of fungal pathogens with high rates of toxicity against cereal crops that can result in crop loss, and if consumed can result in a wide array of diseases in livestock [145,146]. Fusaric acid, in particular, can inhibit the production of dopamine-beta-hydroxylase, which acts as a messenger of signals within the nervous system and is responsible for altering the enzyme tyrosine hydrolase, which is involved in a rate-limiting step in catecholamine synthesis [147,148,149]. Zinc perchlorate Zn(ClO_4_)_2_ and zinc sulfate (ZnSO_4_) also inhibit mycelial growth that produces mycotoxins and reduces the production of mycotoxins themselves [150,151].

#### 2.1.1. Zinc Transport and Homeostasis

Many fungi have mechanisms of zinc transport similar to that of other eukaryotes, through the ZRT (zinc regulated transporter)-IRT (iron-regulated transporter)-like protein (ZIP) family and the cation diffusor facilitator (CDF) protein family [152,153]. In *S. cerevisiae,* zinc transport occurs through several protein groups; the ZIP protein family (via Zrt1, Zrt2, and Zrt3), the CDF protein family (via Zrc1, Cot1, and Msc2), the ferrous transport protein Fet4, and others (Figure 1) [105,107,108,133,154,155]. Zrt1 and Zrt2 are high and low-affinity plasma membrane transporters, respectively; both *ZRT1* and *ZRT2* are upregulated in zinc-deficient conditions and repressed when zinc conditions are favorable [15,22,156]. In an excess-zinc environment, Zrc1 and Cot1 mediate zinc transport from the cytosol into the vacuole to prevent toxicity [20,28]. In a zinc-limiting environment, zinc is released back into the cytosol from the vacuole via Zrt3 or is scavenged by zincophore Zps1 [106,157,158]. Zap1 regulates the transcription of *ZPS1* and contains two activators, Ad1 and Ad2, either independently activated or inactivated by the direct binding of zinc ions [105,108,159,160]. These mechanisms effectively control intracellular zinc uptake and help prevent excess accumulation in *S. cerevisiae*.

In filamentous Ascomycota, such as *Apergillus fumigatus*, genes in the ZIP family (*zrfA*, *zrfB*, *zrfC*, *zrfD*, *zrfE* and *zrfH*) also regulate zinc transport [23,27,161]. *zrfA* and *zrfB*, orthologues of *S. cerevisiae ZRT1* and *ZRT2*, respectively, encode zinc membrane transporters that operate in acidic, low-zinc environments and are activated by transcription factor ZafA [161,162]. Conversely, the *zrfC* gene product is an alkaline zinc transporter activated in high pH, zinc-limiting conditions [23,27]. *zrfD/E/H* are not restricted by pH and can function in either acidic or alkaline environments [23]. In *F. oxysporum*, *zrfA* and *zrfB* are also zinc importers regulated by transcription factor ZafA [163]. During infection, ZafA allows *F. oxysporum* to adapt to a zinc-limiting environment, such as if the host enacts nutritional immunity to deprive it of this essential metal [163]. Basidiomycetes have similar homology. *Ustilago maydis* UmZRT1 and UmZRT2 genes, and *Cryptococcus neoformans* Zip1 and Zip2 are homologous to *S. cerevisiae ZRT1* and *ZRT2*, respectively, with similar transport function [20,24,26]. Similarities also exist in the prevention of zinc over-accumulation. *C. neoformans* Zrc1 is homologous to *S. cerevisiae* Zrc1 and mediates zinc transport into the vacuole to prevent toxicity and decrease zinc sensitivity [20].

Mechanisms of zinc uptake and transport in fungi are mostly conserved through *S. cerevisiae* ZIP proteins and homologs. The next section will discuss how negative homeostatic interventions can result in toxicity.

#### 2.1.2. Zinc Toxicity

Zinc-based antifungal compounds have mechanisms of toxicity that vary between species. Zinc pyrithione (ZPT), is a zinc ionophore often used to treat fungal dandruff caused by *Malassezia* spp. and induces toxicity by increasing cellular zinc uptake [164,165]. ZPT also causes partial mitochondrial malfunction by inhibiting mitochondrial synthesis of iron-sulfur clusters, which are integral in electron transport, respiration, and DNA repair and replication [165,166]. In contrast to *Malassezia* spp., ZPT toxicity in *S. cerevisiae* is not a result of increased zinc uptake, rather of increased copper uptake which overloads homeostatic systems [164,167,168]. ZnO NPs are also being explored for their antifungal properties. In *S. cerevisiae*, ZnO NPs reduce ergosterol biosynthesis which, in turn, increases cellular leakage (up to 24%) and depolarization, reduces cell wall integrity, and increases the occurrence of ROS [83]. In filamentous fungi, mechanisms of toxicity are not well-studied. In ericoid fungi, zinc ions reduced hyphal growth by increasing chitin deposition within the cell wall, preventing hyphal extension; zinc also increases hyphal branching and apical swelling, resulting in atypical hyphal morphology [88]. In the molds, excessive zinc exposure reduces hyphal growth, alters hyphal morphology and interrupts conidia and conidiophore development, limiting reproductive capabilities [87]. Zinc sensitivity can aid in the reduction of fungal pests; however, the development of tolerance and resistance can be an impedance.

#### 2.1.3. Zinc Tolerance and Resistance

High-zinc environments can be detrimental to fungi; therefore they must possess resistance mechanisms to overcome toxicity. In yeast, resistance relies on the upregulation of Zrc1 and Cot1, which sequester Zn^2+^ to the vacuole (up to 100 mM) in *S. cerevisiae*, or the endoplasmic reticulum in *C. albicans* (Zrc1) [105,106,107,108,169]. Khouja et al. also described a resistance mechanism via OmFET in *S. cerevisiae*, though it is not yet fully understood [170]. They suggest that OmFET plays a role in Zn^2+^ uptake, and in that role increases tolerance through interactions with Mg, where Mg competes with Zn^2+^ for uptake, increasing intracellular Mg and restricting Zn [170]. In filamentous fungi, zinc resistance is not only attributed to vacuolar sequestration, but also to storage in the cytoplasm, storage in cell walls of spores and hyphae, and cellular efflux; and in ectomycorrhizal fungi, the presence of metallothionein-like peptides confers Zn^2+^ resistance [110,171,172,173]. To further investigate how fungi cope with toxic levels of other micronutrient metals, this review also assessed cellular interactions with copper.

### 2.2. Copper

Copper is also a transition metal and presents itself in oxidation states copper(I), Cu^+^, and copper(II), Cu^2+^ [32,48]. It is essential to agriculture and human medicine where it can serve as a fungicidal or fungistatic agent, or be the determining factor for virulence [174,175]. Some fungal pathogens heavily rely on copper exporters to prevent host-enacted copper toxicity or import machinery to maintain virulence. In both clinical and agricultural settings, fungal exposure to excess copper can result in ionic imbalance. Therefore, homeostatic mechanisms to maintain healthy intracellular copper levels are critical.

#### 2.2.1. Copper Transport and Homeostasis

Generally, copper cannot permeate the plasma membrane and requires membrane transporters for uptake [32,48]. Before internalization, copper must exist as Cu^+^ (cuprous oxide); however, in the environment, it often exists as Cu^2+^ (cupric oxide) and must undergo reduction. In *S. cerevisiae*, cupric reductase Fre1, transcribed by Mac1, reduces Cu^2+^ to Cu^+^, making it readily available for uptake via high–affinity membrane transporters of the copper transporter (Ctr) protein family, Ctr1 and Ctr3 or low-affinity copper transporter Fet4 (Figure 2) [30,31,32,33,176]. Transcription of *CTR1* and *CTR3* is also regulated by transcription factor Mac1, which regulates transcription based on copper availability; copper depletion results in the upregulation of *CTR1*/*3,* and copper repletion results in downregulation [30,177].

After uptake, Cu^+^ serves as enzymatic cofactors. Apoproteins within the secretory pathway require copper for proper functioning, such as the multicopper oxidase Fet3, which is necessary for ferrous iron, Fe(II), uptake, and oxidation [53,178,179,180]. *FET3* is regulated by transcription factor Aft1 (activator of ferrous transport) in iron-deficient conditions and its gene product contains four Cu^+^ binding sites where copper serves as a cofactor for enzyme activation [53,178,181]. Unmetalated Fet3 reduces cell growth in iron-limiting conditions, demonstrating the importance of copper transport [44,182].

Another enzyme dependent on copper is the cytoplasmic Cu/Zn superoxide dismutase (Sod1). This is an antioxidant for superoxide anions (O_2_^•−^) [183,184]. O_2_^•−^ are ROS that cause cellular damage and toxicity and must be effectively dismutated to prevent stress; therefore, delivery of copper to Sod1 is critical [45,185,186]. In *S. cerevisiae*, the cytosolic copper chaperone Lys7 acquires Cu^+^ and delivers it to Sod1, with high specificity [45]. Once Sod1 is metalated, it is then able to catalyze the dismutation reaction that results in O_2_^•−^ being successfully detoxified to hydrogen peroxide (H_2_O_2_) and molecular oxygen (O_2_); H_2_O_2_ is now readily available for further detoxification to water via catalyst Cct1 [183,187,188]. Cu^+^ transport to MTs Cup1 (also known as Cup1-1 and Cup1-2) and Crs5 is also integral to cellular detoxification [18,189]. Both MTs are regulated by transcription factor Ace1 (also known as Cup2), which activates the transcription of *CUP1* and *CRS5* at elevated copper concentrations [167,189]. Cup1 and Crs5 contain 8 and 11-12 Cu^+^ binding sites, respectively, and are responsible for buffering cytosolic copper to maintain safe intracellular copper concentrations [189,190,191]. Though Crs5 has a greater copper binding capacity, it plays a much smaller role in detoxification due to its promoter region, which only has one recognition sequence, compared to four in *CUP1* [189,190,191].

*S. pombe* follows a pattern of copper transport similar to *S. cerevisiae.* Extracellular Cu^2+^ is reduced to Cu^+^ by cell surface reductases before uptake [34,36]. Cu^+^ can then be transported across the cell membrane, depending on the current cell cycle [34,35,36]. During mitosis, an integral membrane complex composed of proteins Ctr4 and Ctr5 are responsible for Cu^+^ uptake, and during meiosis, Mfc1 (localized in the forespore membrane) is responsible [34,35,36]. Expression of ctr4^+^ and ctr5^+^ is regulated by transcription factor Cuf1, and expression of mfc1^+^ is regulated by transcription factor Mca1, both of which are activated or deactivated by the absence or presence of sufficient copper levels, respectively [34,36]. Once inside the cell, copper chaperones such as Cox17, Pccs, and Atx1 transport Cu^+^ to respective organelles [46]. Pccs is a four domain, cytosolic chaperone. The first three domains are responsible for transporting Cu^+^ to unmetalated Sod1 in a copper-limited environment, activating Sod1 [46]. In high copper environments, the fourth domain acts as a copper buffering system, sequestering Cu^+^ to prevent toxic cytosolic levels [46]. Atx1 in *S. pombe* plays a similar role to Atx1 in *S. cerevisiae*. In *S. pombe*, Atx1 is also located in the cytosol and responsible for carrying Cu^+^ to Ccc2 [34,42]. Peter et al. and Beaudoin et al. described how Atx1 was also used for copper transport to copper amine oxidases (CAOs), a group of catalysts not present in *S. cerevisiae* [34,42]. Atx1 shuttles Cu^+^ to an active site on the CAO, where copper (and another required cofactor, 2, 4, 5-trihydroxyphenylalanine quinone) activates it [34,42,192]. *S. pombe*‘*s* Cox17 is an orthologue to *S. cerevisiae* Cox17, sharing 38% identity and is located in the mitochondrial intermembrane space [42,48]. Once Cox17 acquires Cu^+^ it is delivered to Sco1, Sco2, and Cox11 for copper loading to cytochrome c oxidase subunits [42,47,48].

Filamentous fungi are also important in assessing copper homeostasis, as these organisms depend on copper for growth and virulence in pathogenic species. In the pathogenic Ascomycete *Aspergillus fumigatus*, studies have shown similarities to *S. cerevisiae* and *S. pombe* in copper uptake. Cu^2+^ must also be reduced before uptake, however, there is some ambiguity regarding the reductases responsible [39]. This reductase has been referred to as unknown ferric reductase (“Fre?”), a general Fre reductase, and metallo-reductase Afu8g01310 (homolog of *S. cerevisiae FRE* or *FRE3*) [39,193,194]. After reduction, CtrA2 and CtrC (both homologs of *S. cerevisiae* Ctr1) transport Cu^+^ into the cytosol and serve as enzymatic cofactors [37,39]. CtrA2 and CtrC are regulated by transcription factor MacA (also referred to as AfMac1) which senses low copper concentrations and activates CtrA2 and CtrC [39,49,195,196]. Conversely, in high copper concentrations, transcription factor AceA activates P-type ATPase CrpA as a defense mechanism for copper export and is responsible for extended life and virulence [39,49,195,196].

Limited knowledge exists on copper homeostasis in Basidiomycetes. Studies in two Basidiomycetes, the brow-rot fungus *Fibroporia radiculosa* and the edible white-rot fungus *Pleurotus ostreatus*, reported some details. In *F. radiculosa*, only the regulation of intracellular Cu^+^ concentration has been unveiled, by three, unnamed copper ATPases and one gene of unknown function, CutC, [197]. In *P. ostreatus*, membrane protein Ctr1 is involved in copper uptake and shares homology with the low-affinity copper transporter PaCtr2 of the Ascomycete *Podospora anserine* (20%) and the high-affinity *S. cerevisiae* copper transporter, Ctr1 (20%) [38]. This review shows that copper homeostasis is well-studied in *S. cerevisiae* and *S. pombe*; however, more research is needed in other Ascomycetes and Basidiomycetes.

#### 2.2.2. Copper Toxicity

Copper contains antifungals that have been investigated against various fungi. In *S. cerevisiae*, cupric sulfate (CuSO_4_) and copper oxide nanoparticles (CuO NPs) significantly reduce growth in a dose dependent manner, with the toxicity of both potentially related to Cup2 [113,198,199]. Deletion of *CUP2* increases copper sensitivity, suggesting that a mechanism of toxicity could be reducing or inactivating its regulation, resulting in decreased Cu^+^/MT binding and increased cytosolic Cu^+^ [200,201]. Giannousi et al. found that CuO NPs cause DNA damage that interferes with replication and increases lipid peroxidation, reducing membrane lipid content, resulting in porous cells [202]. In *Candida* spp., CuO NPs have also shown toxic capabilities by inducing porous cell membranes [12]. Copper(II) complexes which have been shown to exhibit fungicidal and fungistatic activity in species that have a history of azole resistance appear to have a similar mechanism by reducing ergosterol content [203,204,205,206,207]. In filamentous fungi, copper also has dose-dependent toxicity. In the agricultural pathogen *Rhizoctonia solani*, a copper (II)–lignin hybrid had high efficacy and significantly reduced the number of plants attacked by *R. solani* [84,208]. In some instances, fungi can overcome toxicity by increasing their tolerance, which may be beneficial in the case of nanoparticle production, but can become a nuisance in pathogenic species.

#### 2.2.3. Copper Tolerance and Resistance

Since copper is implicated as an antifungal agent, its ability to evade copper toxicity must be continuously evaluated. In *S. cerevisiae*, short-term exposure to CuSO_4_ causes significant regulation of open reading frames (ORFs) responsible for cellular detoxification and Cu^+^ uptake [113]. Exposure results in the upregulation of *CUP1* (~20-fold,) and *CRS5* (~8-fold) and the downregulation of *FRE1, FRE7,* and *CTR1* (0.07, 0.08, and 0.10-fold, respectively) [113]. This fold change, and increased CuSO_4_ sensitivity in *cup2*Δ mutants indicates MTs, coupled with decreased Cu^2+^ reduction and decreased Cu^+^ uptake, are likely to be employed as mechanisms of copper resistance [113,200].

Less is known about copper resistance in filamentous fungi. In *Aspergillus* spp., P-type ATPase CrpA has Cu^+^ exporting activity that aids in cellular detoxification, increasing Cu^+^ resistance [90,193,209]. High-affinity copper importers, CtrA2 and CtrC, may be involved in resistance, but are still under investigation [37,49]. In *Fusarium graminearum*, copper exposure upregulates *FgCrpA* (ATPase exporter) and the MT *FgCrdA* as a means to prevent over accumulation, with the predominate method being Cu^+^ export activity [14]. In *F. oxysporum*, upregulation of oxidoreductase activity may decrease susceptibility to oxidative stress that can be induced by excessive copper exposure [210]. In Basidiomycetes, some progress has been made in identifying resistance mechanisms in *F. radiculosa*, where increased production of copper oxalate increases resistance [119]. However, this is the extent of the knowledge.

### 2.3. Iron

Iron (Fe) is a transition metal belonging to group eight of the periodic table and can exist as ferrous (Fe^2+^) or ferric (Fe^3+^) iron [211,212]. As an essential nutrient, Fe is significant for the virulence of fungi that cause disease. In *A. fumigatus* and *F. oxysporum*, survival depends on the ability to sequester iron from the host and a well-functioning homeostatic system to maintain this delicate balance [213,214]. Incapacitating the ability to do so reduces virulence and becomes a growth limiting factor, such as in the use of excessive amounts of Fe to completely overrun homeostatic systems [215,216,217]. Thus, homeostatic mechanisms are integral.

#### 2.3.1. Iron Transport and Homeostasis

Generally, in *S. cerevisiae*, two iron uptake systems are described, the reductive and nonreductive systems. The reductive system recognizes Fe^2+^ salts and chelates for uptake through importers, while the nonreductive systems utilizes iron siderophores [218,219,220,221]. In the reductive system, high-affinity (aerobic) and low-affinity (anaerobic) transporters are responsible for ferric and ferrous iron transport, respectively [222,223]. For low-affinity uptake, iron must be reduced by ferric reductases Fre1 or Fre2, initially described by Lesuisse et al. in 1987 and later coined Fre1 and Fre2 by Georgatsou and Alexandrakin in 1994 [221,224]. Since then, both metallo-reductases have also been found to reduce both cupric and ferric ions, where *FRE1* expression induces the reduction of Cu^2+^ when transcription factor Mac1 is bound, and Fe^3+^ reduction occurs via binding of transcription factor Aft1 [176,181,225]. After Fe^3+^ reduction, Fe^2+^ is then ready for uptake by a six domain, transmembrane, metal transporter, Fet4 [54,55]. Fet4 can also import other metals, but is mostly responsible for Fe^2+^ uptake in iron-restricted cells [223]. In anaerobic conditions, transcription factor Aft1 is required for activation, and in aerobic conditions, expression of *FET4* is repressed by Rox1, which has two binding sites in the *FET4* promoter region [223]. This repression is necessary to prevent the unintended uptake of toxic metals, such as Cd, where it is demonstrated that *rox1*Δ mutants have increased sensitivity to Cd under aerobic conditions [223,226]. A second, less utilized iron transporter in the low-affinity uptake system is Smf1, responsible for the uptake of the Fe^2+^/H complex [51,52]. This metal transporter is mostly known for the uptake of Cu, Mn, and Cd; however, in a study completed by Cohen et al. in 2000, it was shown that overexpression of *SMF1* also results in significant iron uptake [52,65,227]. High-affinity iron uptake is also part of the reductive system. In low-iron conditions, this system dissociates and reduces ferric iron, via Fre1 and Fre2, from a wide array of Fe^3+^ substrates such as ferric chelates, salts, and siderophores [218,219]. Fe^2+^ then transitions through the Fet3/Ftr1 complex [58]. Fet3 is activated by transcription factor Aft1 in iron-deficient conditions and contains four Cu^+^ binding domains that must be metalated for activation [53,178,181]. Activated Fet3 goes through an aerobic reaction that oxidizes Fe^2+^ to Fe^3+^ for passage to the cytosol via iron permease Ftr1 [58,178]. The final destination and the cell’s utilization of Fe^3+^ is not fully elucidated. 

The nonreductive system utilizes siderophores. *S. cerevisiae* is incapable of producing siderophores, but can sequester siderophores produced by other microorganisms via siderophore iron plasma membrane transporters Arn1—Arn4 [16,224]. Arn1 transports ferrichrome into the cell for iron acquisition; however, Arn1 is not always readily available in the plasma membrane because it is localized to endosomes or is routed to vacuoles for degradation when ferrichrome is unavailable [16,220,228]. When ferrichrome is present, Arn1 is routed through the plasma membrane, where ferrichrome adheres to either the low or high-affinity binding site and is transported to the cytosol [16,220,228]. It remains intact in the cytosol and serves as an intracellular Fe^3+^ storage reservoir until the cell needs iron; in this event, Fe^3+^ is reduced via metallo-reductases, or released via ferrichrome degradation [16,220,228,229]. Arn2 (also known as Taf1) is the second siderophore transporter in the *ARN* family, responsible for transporting tri-acetyl-fusarinine to the cytosol; it is unclear if Arn2 is located anywhere else aside from the plasma membrane when tri-acetyl-fusarinine is unavailable [220,230,231]. The literature is not very informative on the functions of tri-acetyl-fusarinine, but it does appear to have a similar role to ferrichrome as a store reservoir for ferric iron [230,231]. Arn3 (also known as Sit1) is a transporter for ferrioxamine B and is situated within intracellular vesicles. It appears to have a similar function to Arn1 and can progress to the plasma membrane when ferrioxamine B is available [229,232]. After ferrioxamine B is transported inside the cell, it is stored in the vacuole, likely for subsequent dissociation [232]. The first three mentioned siderophores transported by Arn1–Arn3 belong to the hydroxamate class of siderophores. However, the final transporter Arn4 (also known as Enb1) transports a siderophore of the catecholate class, ferric entero-bactin [220,233]. Unlike the other siderophore transporters, Arn4 remains at the plasma membrane regardless of the presence of its substrate [218]. Philpott and Protchenko suggested the difference in plasma membrane cycling between hydroxamate and catecholate transporters may be due to the possibility that there are toxins that can adhere to the hydroxamate transporters and not the catecholate transporters [218]. In the act of self-preservation, those transporters remove themselves as a potential source of toxicity [218]. Ferric entero-bactin is not well-studied in *S. cerevisiae*, but based on the function of other siderophores it may be reasonable to conclude that, upon cellular entry, ferric entero-bactin is also used as an Fe^3+^ storage system.

After Fe uptake, there are many intracellular destinations. Two briefly discussed here are the cytosol and the nucleus [61,62]. In the cytosol, iron–sulfur assembly (CIA) proteins Npb35 (binds two Fe–S clusters), Nar1, Cfd1 (binds one Fe–S cluster), and Cia1 form an iron–sulfur complex [61,62,234]. These complexes transfer Fe–S clusters to various apoproteins for activation [61,62,234]. In the nucleus, CIA proteins deliver Fe–S clusters to various nuclear proteins involved in DNA repair and replication [61,235].

Iron homeostasis in the fission yeast *S. pombe* is also well-studied and has three mechanisms of iron uptake [236]. One involves cell surface ferric reduction, and the other, in contrast to *S. cerevisiae*, involves the production of siderophores to capture extracellular iron and heme [236]. The first iron uptake system described here is through use of siderophore synthesis [237]. Under iron-deficient conditions, Sib2, a catalyst for ferrichrome synthesis, hydroxylates ornithine to N^5^-hydroxyornithine, a newly formed hydroxy-mate group molecule, and then undergoes processing by Sib1 [236,237]. This non-ribosomal peptide synthase yields the desferri-form of ferrichrome [236,237]. Schrettl, Winkelmann, and Haas suggested that the resulting ferrichrome is excreted from the cell to capture extracellular Fe^3+^ from the surrounding environment [237]. In an iron-dependent response, transcription factor Fep1 activates ferrichrome transporters Str1, Str2, and Str3, and the iron-loaded ferrichrome re-enters the cell (predominately by way of Str1) [59,63]. *S. pombe* is also able to import exogenous iron-loaded ferrioxamine B via Str2 [63]. In addition to the previously mentioned siderophore functions, it had also been suggested that, as in *S. cerevisiae*, imported siderophores also serve as iron storage vesicles [63,237].

The second iron uptake mechanism employed by *S. pombe* is the high-affinity, reductive system that depends on cell surface ferric reductase Frp1. *frp1*^+^ shares 27% homology with the *S. cerevisiae* Fe^3+^/Cu^2+^ reductase encoding gene, *FRE1*, and reduces extracellular Fe^3+^ to Fe^2+^ [238]. Transcription of *frp1*^+^ may also have some functional relation to the vacuole/cytoplasmic transporter Abc3 that transports iron from the vacuole to the cytosol in iron-deficient conditions [238,239]. Pouliot et al. found that *abc3*Δ mutants resulted in the activation of *frp1*^+^; however, a nucleotide-based transcription factor directly linked to *frp1*^+^ has not yet been determined and it appears to be solely activated or repressed by the absence or presence of iron, respectively [238,239]. After ferric reduction, Fe^2+^ enters an oxidase-permease complex, similar to that of the *S. cerevisiae* Fet3/Ftr1 complex, composed of proteins Fio1 and Fip1 [50]. Fio1 is a Fe^2+^ oxidase that shares 37% homology with the *S. cerevisiae* Fet3, and in an iron deprived environment, oxidizes Fe^2+^ in preparation for transfer across the plasma membrane via Fip1 [50]. fip1^+^ is a ferrous permease having 46% homology with the *S. cerevisiae* Ftr1 [50,236].

Heme is an iron-containing compound and its acquisition and biosynthesis are the finally discussed mechanisms of iron uptake in *S. pombe*. It is notable to state that while *S. cerevisiae* does utilize heme in other processes such as respiration and ergosterol biosynthesis, it has not been determined to be used to acquire iron [240,241]. *S. pombe* imports exogenous heme for iron uptake through Str3 and Shu1 [56,57]. Shu1 is a plasma membrane protein induced during iron deprivation, when heme biosynthesis is not attainable, or if Fep1 is inactivated [56,57]. The second protein involved in heme uptake is Str3, previously mentioned as a part of a ferrichrome transporter family (Str1, Str2, and Str3). Str3 shares the lowest homology (25.1%) with Str1 when compared to Str2 (29%), and its substrate specificity is undetermined [57,59,63]. Iron release and utilization from heme is not yet fully understood in *S. pombe*; however, studies in *C. albicans* (and other fungi) show that heme degradation is catalyzed upon cellular entry via heme oxygenase [56,57,242]. *S. pombe* also biosynthesizes heme and is encoded by *hem1*^+^, *hem2*^+^, *hem3*^+^, *hem12*^+^, *hem13*^+^, *hem14*^+^, *hem15*^+^, and *ups1*^+^ [56,57]. In iron-deficient conditions, a cascade of events between the mitochondria and the cytoplasm occurs to synthesize heme for further utilization [56,57,243].

In addition to iron acquisition in *S. pombe*, regulation mechanisms must be in place to prevent over-accumulation. Mercier, Pelletier, and Labbé identified the gene *pcl1*^+^ to play a role in vacuolar iron storage [244]. *pcl1*^+^ shares homology to *S. cerevisiae* Ccc1, an iron vacuolar transporter, and it has been shown that *pcl1*Δ mutants have increased sensitivity to iron; this together with the study of Mercier, Pelletier, and Labbé suggests that Pcl1 might play a similar role in iron storage in *S. pombe* [63,239]. As mentioned, the final destinations of heme are somewhat unclear, but based on research in other fungi, heme may be degraded, and literature suggested that there may be a group of proteins responsible for transporting those ions to the vacuole for degradation or storage [56,57,245]. Much is known about iron homeostasis in *S. pombe;* however, there are apparent gaps in knowledge of specific processes.

In filamentous fungi, iron homeostasis is less documented. It has been investigated in *U. maydis*, a pathogenic fungus that causes corn smut disease and whose virulence is associated with iron acquisition [60,121]. There are two iron uptake mechanisms, one through hydroxamate siderophores, and the other an oxidase-permease system, similar to *S. pombe* [60,233,246,247]. In the latter, exogenous ferric iron is reduced by a seemingly unknown reductase (possibly Fer9) and then re-oxidized by ferroxidase Fer1 for uptake through the high-affinity ferric iron permease Fer2 [60,121]. In the former, siderophore iron uptake is mediated by siderophore biosynthesis encoding genes *Sdi1* and *Sid2*, and both are negatively regulated by transcription factor Urbs1 [60,121,246,247]. These siderophores play a role in iron acquisition; however, deletion mutants showed they are not necessary for virulence [121].

#### 2.3.2. Iron Toxicity

Iron is involved in many biological processes, but can be toxic in excess. Studies have shown its toxicity in *S. cerevisiae* and fungal pathogens, but they have also demonstrated that targeting and interfering in iron acquisition mechanisms can also be detrimental. Reports indicate that iron or iron compounds are fungistatic against *F. oxysporum* and its mycotoxins in a dose dependent manner [216,217]. In discussing iron toxicity, it is also important to note that the interference of homeostatic systems can result in the inhibition of iron acquisition, which can also be toxic. Leal et al. demonstrated this with the utilization of lactoferrin, an iron-binding glycoprotein, as a topical agent to obstruct iron uptake mechanisms of *A. fumigatus* and *F. oxysporum* in mice [92,93]. Results indicated that, during corneal fungal infection, these fungi acquired iron through siderophores and that the iron-binding agent blocked the ability of the pathogen to acquire siderophore-bound iron, highlighting the inability of the fungi to proliferate without access to iron [93]. In *S. cerevisiae*, iron toxicity is related to the ability of the cell to transport cytosolic iron to the vacuole via Ccc1 [91,248]. Lin et al. showed that *ccc1*Δ mutants could not transfer cytosolic iron to the vacuole under anaerobic conditions, even with the overexpression of iron mitochondrial transporter Mrs3, effectively inducing toxicity [248]. The ability to alter and control homeostatic mechanisms are determinants of the fungal ability to resist excessive iron concentrations.

#### 2.3.3. Iron Tolerance and Resistance

*S. cerevisiae* achieves iron resistance through the downregulation of iron import systems via Aft1, or activation of vacuolar transporter Ccc1 [64,249]. Ccc1 is regulated by the iron sensitive transcription factor Yap5; removal of *YAP5* increases iron sensitivity, while its overexpression dramatically reduces cytosolic iron [120]. It may be worth the effort to investigate how the overexpression of *CCC1* affects iron resistance and the vacuolar ability to store excess iron in order to prevent toxicity. Another vacuolar gene, *VMA13*, might also play a potentially novel role in iron tolerance [250]. Vma13 is commonly known as a vacuolar H^+^-ATPase subunit that plays a role in vacuolar acidification; however, a study involving *vma13*Δ mutants showed that they experienced increased sensitivity to iron deprivation, suggesting Vma13 plays a role in iron import [250]. The function of *VMA13* in iron homeostasis combined with its role in vacuolar acidification should be studied to determine if mutants can also help increase iron resistance. Another method of iron resistance in *S. cerevisiae* is the expression of ferritin related genes. Ferritin is an iron storage protein found in many other eukaryotes, but is not native to fungi [122,123,124]. Its effects on increased iron resistance and storage capacity in yeast has been investigated and results indicate that the expression of human, soybean, and tadpole ferritin genes (*HuFH*, SFerH1/SFerH2, and *TFH*, respectively) resulted in the increased ability of yeast to store and carry higher concentration of iron [122,123,124]. Llanos et al. showed the ability of soybean ferritin genes, SFerH1 and SFerH2, to increase iron resistance in *ccc1*Δ mutants [122]. This is significant because, even without the natural vacuolar detoxification system, yeast cells with soybean ferritin were still able to store increased concentrations of iron and evade toxicity.

Fewer studies report on iron resistance in other fungi, but several inferences can be made based on knowledge of iron homeostasis. In *S. pombe*, ferrichome production, excretion, and subsequent uptake are used to acquire extracellular Fe^3+^ in iron-deficient conditions [59,63,236,237,251]. The engineering of cells to overexpress Sib2 and Sib1 could potentially serve as extra storage vesicles for any excess cytosolic iron acquired by the cell [59,63,236,237,251]. It is not clear how ferrichrome is excreted from the cell after production, therefore this exact mechanism would first need to be identified and well-studied to determine if inhibiting excretion would have any other adverse effects on cellular health. *U. maydis* also biosynthesizes siderophores (hydroxamate) via *sid1* for iron uptake which could also be investigated for increased production for storage of excess iron [60,121].

### 2.4. Manganese

Manganese (Mn) is a transition metal and also an essential micronutrient in fungi. In agriculture, Mn compounds reduce mycelial growth of fungal pathogens [252,253]. In other pathogenic fungi, Mn^2+^ is required for virulence [254]. Some lignocellulose degrading enzymes also require Mn^2+^, such as manganese-dependent peroxidase, which white-rot fungi express during lignocellulose degradation, integral to nutrient uptake [255,256]. Many fungal species rely on Mn^2+^ and homeostatic mechanisms must exist to ensure proliferation.

#### 2.4.1. Manganese Transport and Homeostasis

Within *S. cerevisiae*, Mn^2+^ transporters Smf1 and Smf2 (part of the Nramp metal transporter family) and phosphate transporter Pho84, have a diverging consensus on their roles in Mn^2+^ homeostasis. In the case of Smf1, it was initially determined to be a high-affinity plasma membrane transporter, which acquired extracellular Mn^2+^ in Mn^2+^ deficient environments [65,66]. Smf2 is localized in golgi-like vesicles and shares approximately 50% identity with Smf1 (at the amino acid level), but does not share functionality and is a low-affinity Mn^2+^ transporter [77,257]. Once inside the cell, the fate of Mn^2+^ is as a cofactor for proteins such as Sod2 [126,258]. Sod2 is a mitochondrial manganese superoxide dismutase that receives Mn^2+^ via Mtm1 for activation [69,126,258,259]. In *smf2*Δ mutants, the Sod2 primary protein structure accumulates in the mitochondria; however, they were mostly inactive due to inadequate Mn^2+^ transfer to the mitochondria, indicating that Smf2 is a requirement for *S. cerevisiae* Sod2 activity [126,258]. Smf1 and Smf2, unlike many other metal ion transporters discussed in this review, are not regulated at the transcriptional level, rather post-translationally by protein turnover and localization, which is directly related to Mn^2+^ availability [260]. When Mn^2+^ concentrations are stable or in excess (~100 nmol/(1 × 10^9^ cells)), Smf1 and Smf2 are ubiquitinated via Rsp5 (a NEDD4 family E3 ubiquitin ligase) with the aid of Bsd2 and transferrin receptor-like proteins (Tre1 and Tre2) [260,261,262]. Smf1 and Smf2 are then trafficked to multivesicular bodies, which deliver the proteins to the vacuole for degradation [260,263,264]. This mechanism of action is supported by reports that *tre1*Δ, *tre2*Δ, and *bsd2*Δ mutants resulted in the accumulation of Smf1 and Smf2 [260,261,262]. Conversely, when Mn^2+^ starvation occurs, Bsd2 is depleted, Smf1 is localized to the cell surface, Smf2 is localized to intracellular vesicles, and Smf1 and Smf2 resume their Mn^2+^ uptake functions [257,260,261].

The final transport system discussed here is the phosphate transporter Pho84. It was initially characterized in *S. cerevisiae* as a high-affinity, six-domain, transmembrane, inorganic phosphate transporter [265]. However, Pho84 is now also known as a low-affinity Mn^2+^ transporter, along with other metals such as cobalt, zinc, and copper [67]. Through *pho84*Δ mutants, it was shown that Mn^2+^ uptake was the most commonly affected (in relation to the other metals) when *PHO84* was removed, further proving its Mn^2+^ transporter role [67]. *PHO84* transcription is regulated by transcription factor Pho4, which inhibits Pho84 activity when it is phosphorylated in the presence of excess phosphate; Pho4 resumes transcription when phosphate levels are low [265,266].

Once Mn^2+^ is inside the cell, there are an array of destinations. Pmr1 (high-affinity Ca^2+^/Mn^2+^ P-type ATPase) and Gdt1 (calcium/manganese transporter) both transport cytosolic Mn^2+^ to the Golgi lumen, where Mn^2+^ serves as a cofactor for mannosyl-transferases, such as Mnn1, Mnn2, Mnn5, and Mnn9, which glycosylate proteins in the secretory pathway [70,71,72,267,268,269,270,271]. This type of protein modification provides protein stability by preventing degradation, protecting against oxidative damage, and increasing thermodynamic equilibrium [272]. Concerning the ER, P-type ATPase Spf1 transports Mn^2+^ to the ER lumen; this is supported by a study showing that *spf1*Δ mutants had decreased luminal Mn^2+^; its overexpression had the opposite effect [73]. This same study also stated that Mn^2+^ depletion observed in *spf1*Δ mutants negatively impacted luminal Mn^2+^ dependent processes. On the contrary, it positively impacted Mn^2+^ associated cytosolic processes, indicating that Spf1 is integral to *S. cerevisiae* manganese ER and cytosolic homeostasis [73].

**Figure 3:**
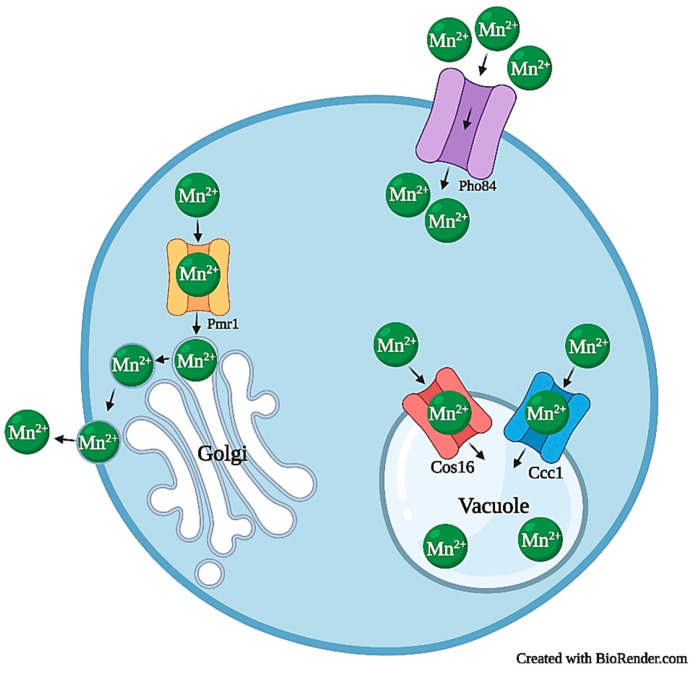
Mn^2+^ uptake and detoxification systems in *S. cerevisiae*.

Mn^2+^ accumulation can have severe consequences on cellular health, and systems must be in place to prevent subsequent events. We will discuss two defense mechanisms in *S. cerevisiae*, Mn^2+^ trafficking to vacuoles for storage and degradation and Mn^2+^ export. Pmr1, previously characterized as an Mn^2+^ Golgi lumen transporter, also serves as a detoxifier. Presented with toxic Mn^2+^ levels, Mn^2+^ is still transported to the Golgi lumen from the cytosol, but excess ions are delivered to secretory pathway vesicles, which ultimately exit the cell, completely removing toxic Mn^2+^ (Figure 3) [77,273]. The *HIP1* gene product also expresses export activity. Hip1 was initially characterized as a high-affinity, plasma membrane histidine permease, but has since been shown to play a role in Mn^2+^ resistance [78,274]. Farcasanu et al. investigated *S. cerevisiae* mutants having defects in Mn^2+^transport and found that a mutation in the *HIP1* gene was responsible [78]. This mutation, originally a single base deletion, introduced a cascade of mutations that led to the protein Hip1-272 (272 amino acids long). Subsequent experiments showed that *hip1-272* mutants had significantly less cytosolic Mn^2+^ accumulation, increased Mn^2+^ efflux, and increased resistance than null mutants and wild type strains [78]. Further studies into the *hip1-272* mutant could elucidate the exact mechanisms of action of Mn^2+^ transport, determining how ions are trafficked to Hip1-272 and expelled. The second defense mechanism against Mn^2+^ toxicity in *S. cerevisiae* was Mn^2+^ trafficking to vacuoles through Ccc1 and Ypk9. Ccc1 (and possibly Cos16) is localized in the vacuolar membrane and is responsible for trafficking cytosolic Mn^2+^ to vacuoles; *CCC1* overexpression results in reduced Mn^2+^ toxicity, lower concentrations of cytosolic Mn^2+^, and increased vacuolar concentrations (Figure 3) [64,75,77]. Ypk9 is also localized in the vacuolar membrane and shuttles Mn^2+^ to the vacuole. Gitler et al. and Schmidt et al. both demonstrated that *ypk9*Δ mutants expressed Mn^2+^ hypersensitivity when compared to wild type strains, further affirming Ypk9 involvement in Mn^2+^ homeostasis [74,76].

Manganese homeostasis has not been well characterized in higher fungi, but *Phanerochaete chrysosporium* has received some attention. *P. chrysosporium* is a white-rot fungus that produces lignin-degrading enzymes, which have been useful in the biodegradation of various plant biomass and an array of organo-pollutants [275,276,277]. Manganese peroxidase is a common lignin depolymerizing peroxidase utilized by white-rot Basidiomycetes [278,279]. It acts in combination with other enzymes to convert various biomass to useful bio-products of commerce and agricultural operations [255,280,281,282,283]. Homologs of the *S. cerevisiae* Pho84 and Smf1/2 proteins have been found in *P. chrysosporium*, PcPho84 and PcSmfs, respectively. PcPho84 is a plasma membrane protein involved in Mn^2+^ uptake, having a similar function to its *S. cerevisiae* homolog [68]. Smf1/2 are predicted to have similar functions in *P. chrysosporium* to their *S. cerevisiae* homologs [68]. Intracellular Mn^2+^ transport has also been investigated. Yeast homolog PcAtx2, localized in the Golgi membrane, was shown to function as an antioxidant through *sod1*Δ mutants [68]. When grown on 600 µM paraquat (inducer of oxidative stress), *sod1*Δ mutants experienced almost no growth; however, in mutants expressing *PcATX2*, growth was restored, indicating that PcAtx2 exhibits similar antioxidant functionality as Sod1 [68]. In the case of mitochondrial transport, *S. cerevisiae* Mtm1 traffics Mn^2+^ to the mitochondria for Sod1 activation; however, the function of the *P. chrysosporium* homolog, PcMtm1 (localized in the mitochondrial membrane), has yet to be identified, but predicted to have a similar antioxidant activity [68]. PcMnt and PcCcc1 engage in Mn^2+^ storage and export in *P. chrysosporium*, respectively. In *Phanerochaete sordida*, PsMnt was found to be a homolog of yeast Smf2 and plays a role in Mn^2+^ uptake, suggesting that it could have dual functionality, but this is still unknown [77,284]. Limited information exists on Mn^2+^ homeostasis in other fungi; however, due to the impact of Mn^2+^ on lignin-degrading enzymes in wood-rotting fungi, more studies should be conducted. Overall, Mn^2+^ homeostasis is critical to cellular functioning to prevent toxic Mn^2+^ accumulation, detoxify cells of free radicals, and provide white-rot fungi with their capacity to degrade lignin. In the absence of such mechanisms, toxicity can impede proper functioning and cause cellular damage.

#### 2.4.2. Manganese Toxicity

In the model yeast *S. cerevisiae*, excessive Mn^2+^ can overrun homeostatic systems and create a toxic ionic imbalance that negatively impacts survival rate [104,285,286]. Expression profiles show that high levels of Mn^2+^ down-regulate genes associated to histidine proteins (*HTB2*, *HTA1*, *HTA2*, *HTB1*, and *HHF*) that are compulsory in chromatin assembly chromosome functioning, and interface in this functioning can end in cell cycle arrest [94,95,114,287]. Filamentous fungi are often studied for their lignin degradation properties which focus on how Mn^2+^ impacts manganese peroxidase activity, but there is a lack of knowledge on how excess Mn^2+^ can be toxic towards this activity [96,97,98]. Due to the reliability of much of the lignin degrading properties on manganese peroxidase in many white-rot fungi, effects of Mn^2+^ over accumulation should be further investigated [96,97,98]. In some cases, toxicity can be avoided by resistance mechanisms.

#### 2.4.3. Manganese Tolerance and Resistance

As with other metals, Mn^2+^ resistance is usually contingent upon homeostatic systems. In *S*. *cerevisiae*, several genes involved in resistance emanate from mutations. *MNR1* (also known as *HUM1* and *VCX1*), encodes a vacuolar H^+^/Ca^2+^ antiporter, but has been implicated in Mn^2+^ resistance [125,288,289]. A single nucleotide alteration may affect Mnr1 function and result in increased Mn^2+^ sequestration to the vacuole [125,289,290]. A mutation in *PHO84* is also implicated in Mn^2+^ resistance, where *pho84*Δ mutants have increased resistance, likely due to the acquired inability to import and accumulate excess Mn^2+^ [67]. In filamentous fungi, Diss et al. elucidated potential resistance mechanisms through *P. chrysosporium,* where it was demonstrated that *PcPho84*Δ mutants increase Mn^2+^ resistance, as well as expression of *PcMNT*, which is likely to engage in Mn^2+^export activity [68]. 

Up to this point, metals that serve as essential nutrients have been reviewed. In recent years, there has been an increase in studies on the usage of metals with no nutritional purpose, but which serve as antimicrobial agents, such as silver. This increase gives cause for further investigation into how these metals are metabolized and their intracellular functioning.

### 2.5. Silver

Silver (Ag) is a transition metal that shares similar properties to other transition metals in groups three through twelve, and closely resembles the properties of Cu and gold (Au) [291,292]. In fungi, silver is implicated in the eradication of pathogens. As part of agricultural research, silver nanoparticles (Ag NPs) and Ag ions (Ag^+^) have demonstrated their ability to control plant pathogens [293,294,295]. As a feed additive, silver has a positive effect on the intestinal microflora, aflatoxins, and mycotoxin absorption in farm animals and in the food industry is used in food packaging for its antimicrobial properties [291,296,297]. Thus, the development of silver as an antimicrobial agent should continue to be investigated, especially on the development of fungal resistance and the impacts on non-target organisms.

#### 2.5.1. Silver Transport and Homeostasis

Silver is a non-essential metal that has no designated cellular receptors or membrane channels for ion uptake. Much of the literature has focused on silver as an antimicrobial agent, but some studies have begun to clarify homeostatic mechanisms [81,114,116,298]. Silver has properties similar to copper, which has initiated the evaluation of copper homeostatic systems to investigate how they may contribute to silver uptake and transport [21,81,114,116,298].

In *S. cerevisiae*, Ctr1, high-affinity Cu^+^ transporter, has been identified as a Ag^+^ importer. This is based on observed reduced Ag^+^ uptake in *ctr1*Δ mutants exposed to low silver concentrations, and transcriptional analysis that shows exposure to Ag NPs upregulates *CTR1* throughout the entire transcriptome [80,81]. The involvement of copper-related genes in Ag^+^ homeostasis was also investigated by Hosiner et al. and Niazi et al.; both found that short-term exposure to silver resulted in increased expression of copper MTs Cup1-1 and Cup1-2, suggesting these MTs sequester Ag^+^ in response to silver stress [114,115]. The competitiveness of Cu^+^ and Ag^+^ for Cup1-1 and Cup1-2 should be further investigated to determine which ion the MTs have a higher affinity for. Other metal ion transporters (Pho84, Fet3, and Smf1) have been investigated for their involvement in Ag^+^ uptake, but results indicate they are not [81].

Once inside the cell, there are not many known Ag^+^ destinations. AgNO_3_ exposure results in Ag^+^ accumulation in the mitochondria, which, in return, reduces Cu^+^ accumulation in the mitochondrial matrix [21]. The direct result of this action is reduced copper-dependent cytochrome *c* oxidase activity, suggesting that cytosolic Ag^+^ is trafficked to the mitochondria via Cu^+^ mitochondrial transporter Pic2, potentially with a higher affinity, which can be toxic to cells by reducing the rate of cellular respiration [21]. No other intracellular destinations have been identified in yeast, and silver homeostasis in filamentous fungi is still unknown.

#### 2.5.2. Silver Toxicity

Efflux systems are integral to cellular homeostasis, preventing the accumulation of toxic compounds within a cell. In *S. cerevisiae*, Ag^+^ uptake can affect these systems, resulting in toxicity. Exposure to Ag^+^ can increase the efflux rate of potassium ions (K^+^) from *S. cerevisiae*, resulting in almost complete K^+^ efflux from the cell. *S. cerevisiae* requires a minimum 30mM K^+^, suggesting those events can be toxic if the ion concentration is not restored [299,300]. Another mechanism of Ag^+^ toxicity is its ability to alter cellular structure [100,103]. Ionic fluids can affect cell membrane integrity of yeast *Yarrowia lipolytica*, reducing the amount of ergosterol, which fluidizes the membrane, and increases internal lateral pressures [100]. Ag^+^ exposure can also deform the cell wall, which is a likely a response to the down-regulation of genes involved in ergosterol synthesis (*ERG3*, *ERG5*, *ERG6*, *ERG11*, *ERG25*, and *ERG28*) in *S. cerevisiae* [80,99]. In the aquatic fungus *Articulospora tetracladia*, transcriptome analysis via RNAseq revealed toxicity of Ag^+^ and Ag NPs may result from interrupted functioning of plasma/organelle membranes and downregulation of genes associated with cellular redox [301]. Silver toxicity has also been studied in other agriculturally relevant processes and it has been determined that AgNO_3_ and Ag NPs can be useful in pathogen control of plant diseases [174,293,295]. It may be worthwhile to investigate silver homeostasis in addressing long-term effects of exposure.

#### 2.5.3. Silver Tolerance and Resistance

The worldwide increase of silver usage makes studies on mechanisms of silver resistance important; presently, few studies have reported on this. *CTR3* is implicated in Ag^+^ resistance after an observed fold increase in its expression in a silver evolved strain of *S. cerevisiae* [116]. Insight into the expression of the Ctr3 transcription factor *MAC1* in the presence of Ag^+^ may clarify its role in resistance. It is possible that MTs Cup1-1 and Cup1-2 are also involved in resistance. It was previously described that exposure to AgNO_3_ and Ag NPs resulted in the increased expression of *CUP1-1* and *CUP1-2*, proposing that the encoded MTs may also bind Ag^+^ and decrease sensitivity [81,114,115]. Similar results were observed in AgNO_3_ exposure, where yeast had increased expression of *CUP1-1* and *CUP1-2* (4.79-fold and 4.71-fold, respectively) in an extended study that resulted in an evolved yeast strain, confirming the potential role of copper MTs in silver resistance [116]. Other Ag^+^ transporters, Pho84, Fet3, and Smf1, were not implicated in Ag^+^ uptake; however, significant down regulation (68.56-fold) of *PHO84* in silver evolved yeast has been observed, which may indicate that Pho84 plays a role in Ag^+^ uptake, and may serve as a mechanism of Ag^+^ resistance [81,116]. The effect of Ag^+^ on genes involved in ergosterol biosynthesis was also investigated in a silver evolved yeast [116]. Results indicated down-regulation of those genes, suggesting that one mechanism of action of resistance against Ag^+^ toxicity could be the ability to inhibit their down regulation [116]. In the filamentous fungus *A. nidulans*, silver induced expression of copper exporter crpA, indicating that it may play a role in silver export and resistance [90]. In *A. tetracladia*, resistance may be due to increased vacuolar function [301]. Overall, there has been some progress made in unveiling silver homeostasis in fungi, mostly by way of *S. cerevisiae*. Due to the increasing silver and Ag NP usage in many aspects of human life, silver–fungal interactions should be further investigated at the molecular level to decipher precise homeostatic and resistance mechanisms.

## 3. Omics and Metal Homeostasis

As the potential for commercial use of antifungal metals increases, so does the need to further investigate fungal homeostasis of essential and non-essential metals. Currently, research in this area is heavily reliant on assay based methods, which can be subjective and ambiguous. In this review, many of the discoveries of homeostatic mechanisms stemmed from the use of deletion libraries, microarrays, and PCR-based methods. This can restrict the scope of the research by only analyzing known genomic or transcriptiomic signatures.

The incorporation of an omics based approach is a resolution to this issue. The most popular omics utilizes bioinformatics to analyze fungal–metal interactions at a nucleotide and protein level, which can reveal novel genes and mutations. In genomics, the entirety of a genome is assessed and compared to others for similarities and differences that can contribute to an organism’s characteristics [302,303]. Transcriptomics relies on RNA sequencing to survey gene expression through fold-changes in transcripts and proteomics assess fold-change in subsequent proteins. In fungi, omics is already incorporated into the identification of characteristics of multi-drug resistance, analysis of genomic divergence based on species origination, some analysis of metal tolerance due to short term exposure, and the analysis of the effects of exposure to non-metal selective pressures [210,301,304,305,306].

Bioinformatics analysis is used to translate omics results via computer programming methods. In nucleotide based omics, DNA or RNA is fragmented into segments or reads prior to sequencing. After sequencing, base calling assigns a nucleotide base to an intensity signal linked to a chromatogram peak and quality control measures are taken to trim reads of adapters used in the sequencing process and trim low quality bases [307]. Next, species that have a reference genome or transcriptome are mapped or aligned to that reference (resequencing). After genomic mapping, variant calling identifies distinctions between the re-sequenced organism and the reference [307]. After transcriptomic mapping, transcripts are quantified and analyzed for differential expression. Species that do not have a reference undergo de novo assembly, which constructs a genome or transcriptome from scratch. De novo assembly utilizes the fragmented reads by overlapping or matching them based on areas of similarity until the entire -ome is constructed [308]. Genome or transcriptome annotation can then be used for further interpretation of the sequencing data. In other omics, molecules produced by an organism are also analyzed and compared to chosen reference samples.

Steps within these bioinformatics pipelines require the use of computational tools written into the command line. Multiple tools with varying parameters exist to complete the same function; however, the user must decide which tools fit their scientific needs. This can result in variation between datasets and across scientific disciplines, based on accepted standards and norms. However, this limitation does not deduct from the vast amounts of data received.

With the increasing affordability of high-throughput omics, organisms can be analyzed at multiple omics levels. This is leading to a more comprehensive understanding of characteristics, especially in fungi where there is limited knowledge of their complexity. This type of research will also illuminate unique features of fungal metal homeostasis, toxicity, and resistance, especially of non-essential metals that are becoming conventional antimicrobial agents.

## 4. Conclusions

Fungal–metal interactions such as the synthesis of nanoparticles and metal used as antifungal agents are on the rise. Studies on metal toxicity and resistance have uncovered preserved homeostatic mechanisms. This review discussed metal homeostasis in various fungi types and has shown that essential metals have designated uptake and transport systems that regulate metal ion balance, mostly through the model organism *S. cerevisiae*. However, there was a significant lack of fundamental knowledge of such mechanisms in filamentous fungi, which play critical roles in nanoparticle biosynthesis and are targets of metal antifungals, further accentuating the need to investigate molecular systems involved in metal homeostasis. Fungal homeostasis of the non-essential metal silver was also highlighted. It showed that homeostatic mechanisms were reliant on existing copper transport systems, but were largely unclear regarding overall cellular processing. There is a need to further investigate other non-essential metals’ cellular homeostasis as their commercial usage increases, due to the current lack of knowledge of future implications. 

## Data Availability

Data sharing not applicable.
